# Model migration and rough edges: British actuaries and the ontologies of modelling

**DOI:** 10.1177/0306312719893465

**Published:** 2019-12-06

**Authors:** Arjen van der Heide

**Affiliations:** Max Planck Institute for the Study of Societies, Germany

**Keywords:** actuarial science, financial economics, model migration, modelling ontologies, models, social studies of finance

## Abstract

The existing literature on modelling provides two main ways of viewing model migration: a modular view, which seeks to decompose models in their constitutive elements, and thus provides a view on what it is that migrates; and a practice-based view, which focuses on modelling as an activity, and understands a model as intricately entangled with its context of use. This article brings together these two sensitivities by focusing on ontologies of modelling. The paper presents a case study of the appropriation of modern finance theory’s ‘no-arbitrage’ models by British actuaries – a process that gradually unfolded at around the turn of the century and led to significant friction within the UK’s insurance industry. We can distinguish two main modelling ontologies: a ‘risk-neutral ontology’, which underpins no-arbitrage models and holds that the value of financial instruments is determined by ‘arbitrage’; and, a ‘real-world ontology’, which assumes that the economic world consists of real probabilities that may be approximated through a combination of archival-statistical methods and expert judgment. The appropriation of the risk-neutral modelling ontology was made possible by the declining legitimacy of actuarial expertise as ‘financial stewards’ of life insurance companies. The risk-neutral modelling ontology provided an ‘objective’ alternative to the traditional actuarial models, which explicitly required actuaries to make ‘prudent’ judgments. Despite the fact that the no-arbitrage modelling was considered an ‘objective’ affair, the valuation models that insurers use today are strongly shaped by political compromises, a result of the ‘rough edges’ of models.

## Introduction

Simulation models are increasingly identified as an important interface between academic research and policymaking (Svetlova and Dirksen, 2014; Van Egmond and Bal, 2011; Van Egmond and Zeiss, 2010). Models can be useful tools for policymaking because they mimic some object of interest, which can then be manipulated to see how it would respond to a variety of exogenous factors. Simulation models, such as climate models or models used for economic forecasting, produce ‘present futures’ (Beckert, 2016) that guide present action by estimating the likelihood of a variable or a set of variables assuming specific values under changing circumstances (Edwards, 2010; Svetlova, 2018; Winsberg, 2010). One consequence of relying on such models in policymaking is that seemingly minor technical details may have large consequences for the formation of expectations, which may cause stakeholders to problematize the technical specifications of models in decision-making. As is by now well-known in science and technology studies (STS), artefacts such as models ‘have politics’ (Winner, 1980), even if what this phrase means is a ‘site for ongoing contestation’ (Brown, 2015: 4).

This article focuses on an important dimension of the politics of modelling: the conditions that give rise to and the consequences of model migration – the displacement of a set of modelling practices from one context of application to another (Bradley and Thébault, 2017; Humphreys, 2002; Knuuttila and Loettgers, 2014). Such model migration, I argue, is characterized by an interesting paradox. For scientific models to have an influence on the world, they have to travel and become part of the world; in so doing, the models themselves are affected, in terms of how they are interpreted, their material manifestations, and how they are used (Morgan, 2012; Svetlova, 2018; Weisberg, 2013).

In this article I contribute to our understanding of the politics of modelling by investigating the migration of a set of modelling practices from modern finance theory and investment banks’ derivatives departments to the modelling departments of UK life insurance companies. The migration of so-called ‘no-arbitrage models’ has had significant consequences for what life insurance companies do, for their function in contemporary capitalism as intermediaries for the redistribution of the financial uncertainty that comes with long lives or premature deaths. Although the appropriation of no-arbitrage models went by largely unnoticed outside the insurance industry, it nevertheless caused significant tension and friction within the industry itself. Actuaries long resisted the use of modern finance theory’s models, but by the end of the 2000s no-arbitrage modelling was the main approach for determining the value of insurers’ future financial commitments, their ‘insurance liabilities’. It changed how insurers viewed problems of value and risk, contributing, for instance, to the repurposing of some of the largest insurance companies in the UK into asset managers, as they earned their money increasingly from investment mediation and less so from insurance-related activities.

The existing literature on modelling provides two possible ways of viewing model migration: a modular view, which seeks to decompose models into their constitutive elements, and thus provides a view on *what* it is that migrates; and a practice-based view, which focuses on modelling as an activity, and understands a model as intricately entangled with its context of use. Here I bring these sensitivities together by focusing on what I call the ‘ontologies of modelling’, a concept that draws from the empirical ontology literature in STS.

I argue that two modelling ontologies can be distinguished: a ‘risk-neutral ontology’, which underpins the no-arbitrage models developed in modern finance theory; and, a ‘real-world ontology’, which assumes that the economic world consists of real probabilities that may be approximated through a combination of archival-statistical methods and expert judgment. The diffusion of no-arbitrage modelling in actuarial practice was not simply an insertion of already existing models into new areas of application, but was tied up with a transformation of the setting in which it was deployed. Whereas actuaries previously enjoyed much discretionary space to decide how an insurer’s liabilities should be valued, with the introduction of no-arbitrage style modelling financial markets became the ultimate source of ‘knowing’ those values – a feature that is baked into the risk-neutral ontology underpinning these models. With the reduced actuarial discretion also came rigidity, which led some aspects of the market-consistent models to become politically controversial. Overall, the new models and the setting in which they were deployed co-evolved: While the introduction of market-consistent models facilitated a shift in the distribution of epistemic authority, the models themselves were shaped by political negotiations, even if the ontology underpinning these models remained the same.

The case study is based on a historical sociology of the changing evaluation practices in UK life insurance. The most important source is a set of 43 oral-history interviews with professionals from both inside and outside the actuarial profession, who have been involved in varying degrees in the events described in the paper. The interviews ranged in length from 50 minutes to 4.5 hours and lasted on average 1.5 hours. Interviews were conducted with: academic actuaries and consultants (both actuaries and non-actuaries) many of whom have been key to the development of the new epistemic machinery; actuaries who have worked or are still working at life insurance offices and who are involved in valuation work; former and current regulators. While most interviewees preferred to remain anonymous, some are mentioned by name. In addition to the interview data, the findings in this article are also based on a large corpus of documents, including consultation papers, speeches held by regulators, regulatory communication documents (newsletters, briefings, etc.), journal articles, reports, insurers’ annual reports and newspaper articles.

In the next section, I briefly discuss the two dominant views on modelling, before outlining what the empirical ontology literature might have to add to this. I also describe the two modelling ontologies in more detail. In the following sections, I analyse the migration of no-arbitrage modelling to the modelling departments of insurance firms. I describe the role that modelling plays in life insurance, how the migration of no-arbitrage models was entangled with a change in the structure of epistemic authority in UK’s life insurance industry, and how the idealizations necessary to construct a model world that renders the valuation problem computationally tractable may constitute the ‘rough edges’ of a modelling ontology. I address the ontological politics of the appropriation of no-arbitrage modelling and discuss some of the consequences of seeing the world in terms of the risk neutral ontology. Finally, I describe how the modelling became subject of politics at the European level, before coming back in the conclusion to the main points listed above.

## Model migration and the ontologies of modelling

### The modular view: Structures, construal and idealization

In what I will refer to as the modular view, models can be decomposed into their different constituent elements. For instance, in one of the more elaborated conceptualizations of models, Weisberg (2013: 24–31, 39–42) distinguishes between a model’s ‘structure’ and its ‘construal’. A model’s structure provides a formalized account of how the different entities in a model relate to and interact with each other. A model’s construal provides the means to interpret it, by assigning meaning to its constitutive elements, defining the model’s intended scope and providing the criteria based on which its usefulness and accuracy can be assessed. Seen from this perspective, modellers may borrow model structures or ‘computational templates’ from neighbouring disciplines, providing them with new interpretations to adapt them to subject-specific phenomena (Bradley and Thébault, 2017; Humphreys, 2002). This sense of model migration involves the translation of the model to solve an already existing problem. Another sense in which models may travel involves the migration of a ‘conceptual framework’ and ‘tools suitable for articulating and developing this framework’ that migrate, perhaps together with a ‘computational template’ or ‘structure’ (Knuuttila and Loettgers, 2014: 283). In this sense, the world that the model purports to describe is re-articulated as a problem that can be addressed by an already existing model.

In the modular view, models are representations of the world that portray the world in an idealized fashion. Weisberg (2013: 98–105) distinguishes three types of idealizations: (1) those intended to construct ‘minimal’ models, which can explain a general phenomenon rather than accurately describing specific instances of that phenomenon; (2) those that fragment the model of a phenomenon into several distinct, simpler models that are nonetheless interconnected; and, perhaps most importantly, (3) those intended to simplify the structure of the model by leaving out non-essential aspects, justified on pragmatic grounds, for instance to render a problem computationally tractable. Idealizations may thus be seen as necessary and even desirable, because they can turn a model into a useful tool for understanding, prediction and calculation. Idealizations may, however, also be construed as constituting what one of my interviewees referred to as the ‘rough edges’ of a particular style of modelling, possibly undermining the usefulness or indeed the perceived veracity of a particular model. The key point here, however, is that in constructing an idealized calculative space, for instance for reasons of mathematical tractability, a model may privilege some aspects of its object over another (Mann, 2017). The modular view of modelling thus conceives of the politics of modelling in terms of visibility: What models make visible and what they obscure matters.

The strength of this perspective is that it allows people more easily to trace the trajectories of models through different settings, and thus we see explicit discussions of ‘model migration’ (Bradley and Thébault, 2017; Humphreys, 2002; Knuuttila and Loettgers, 2014). As scholars in the philosophy of science and STS have increasingly pointed out, however, in focusing on the representational dynamics of modelling, there is a danger of overlooking the question of how a model influences the behaviour of its user (e.g. Knuuttila, 2011; Morrison and Morgan, 1999). There are, in other words, diverse ways in which models may be used, which differ not only between, for instance, science and policy-making, but also within those domains (Svetlova, 2018).

### The practice view: Translation and performance

Practice-oriented scholars tend to focus not so much on conceptualizing models themselves, but rather on the act of modelling. A model has agency not because of its innate features, but because it is entangled in a web of practices in contingent ways (Morgan, 2012; Pickering, 1995; Sismondo, 1999). As such, models do things. They are performative – that is, they do not just describe the world as it is, but they also contribute to its making (Callon, 1998; MacKenzie et al., 2007; MacKenzie and Millo, 2003). Models, in other words, take part in the performance of reality and are, as such, not entirely inconsequential. Modellers, however, typically use models as creative resources, and, as such, models do not necessarily determine action, but shape the conditions of possibility in which action takes place (Svetlova, 2018). In the practice view of models, then, this is what they do: They enact the object that they purport to describe in a specific way, and, in so doing, render certain trajectories of action possible and maybe even logical, while closing down others.

In the practices view, model migration is an essential aspect of how models acquire ‘performative power’ (Svetlova, 2012). If they are to influence the world, they need to travel in the world (Latour, 1987; Van Egmond and Zeiss, 2010). This raises an interesting problem, for if models are to travel, they also need to be fitted into and adapted to local circumstances. Model migration, in other words, might be envisioned as a process of translation, in which both the meanings ascribed to a model and the objects that it purports to describe are re-constituted (Callon, 1986; Sundberg, 2007). The terms in which the epistemic problem is described needs to be aligned with the terms that constitute the world within the model, and this requires the re-description of both.

Practice-oriented scholars often emphasize that there are important differences between the criteria for a good model in different settings. In science, for instance, the main aim of a model is often epistemic; in finance, however, the main of modelling may be different, for instance to make money. In practice, differences between what Svetlova (2018) calls different ‘cultures of model use’ may not be so clear cut, but they are nonetheless important to keep in mind.

The practice-based literature on modelling provides useful tools for thinking about how models may play a role in shaping the world. In so doing, practice-oriented scholars often tend to stress the haphazard nature of modelling (‘modelling is bricolage!’) and the importance of local meanings attributed to it (MacKenzie, 2003; Sundberg, 2007, 2009; Winsberg, 2010). The literature is less clear, however, about what remains stable when models travel. What, in other words, are the sources of constraint that come with a model? How can the migration of a set of models come to discipline the agents that use it?

### Empirical ontology and the deflationary move

While the modular view of models thus provides a useful way of thinking about models as idealized artefacts that may obscure some dimensions of the reality that they purport to describe in order to accentuate others, the practice-based view highlights the role of models in practice. A third possible way of viewing the politics of modelling, I suggest, combines these sensitivities. It similarly focuses on the practice of modelling, but understands it as an ‘enactment’ of a specific ontology. This draws on the ‘empirical ontology’ perspective that in the past two decades has become increasingly prevalent in STS (Law, 2002; Mol, 2002, 2013; Van Heur et al., 2012; Woolgar and Lezaun, 2013).

Although the precise meaning of the ‘ontological turn’ in STS remains open for debate – deliberately, some argue, so as to allow the concept of ontology to be put to good use (Woolgar and Lezaun, 2015) – scholars seem to agree that it is at the very minimum about the multiplication of reality. By turning ontology into an empirical question, scholars in STS have challenged the notion that reality is singular and immutable, that there is no feedback loop between description of the world and the conditions of possibility within the world itself (Barnes, 1983; Mol, 2002). Instead, the world is enacted and brought into being in a multiplicity of ways, and there may be frictions and tensions between the multiplicity of realities. Mol (1999, 2002) argues that the frictions and tensions give rise to ‘ontological politics’, a style of politics that revolves not only around *how* to act upon reality, but also around *which* reality upon which to act. By deflating the notion of ontology, in other words, it becomes a tool to distinguish between different ways of enacting the world and to make visible the frictions that may emerge between them.

The empirical ontology literature has not been without its criticism. If ontologies are inextricably intertwined with the practices through which they are enacted (e.g. Law and Lien, 2012), why do we need to speak of specific ontologies in the first place, rather than to refer to specific clusters of practices or different ways of enacting an object (Sismondo, 2015: 446)? Moreover, ‘there is a kind of arbitrariness to the number of realities that empirical ontology might describe’ (Sismondo, 2015: 445).

I circumvent the problem of arbitrariness by deploying the idiom of empirical ontology to point out not only that insurance contracts may be enacted differently but also that the two clusters of modelling practices central in this paper are often imputed with distinct ontological qualities by actors and analysts alike (Chiapello and Walter, 2016; MacKenzie and Spears, 2014). In some ways, therefore, the concept of modelling ontology as it is deployed here is an actor category, not a category of the analyst. This is a crucial point because the ontological qualities ascribed to the model seem to be important in designating no-arbitrage models as authoritative. One of the reasons for no-arbitrage models to enjoy a great degree of ‘performative power’ (Svetlova, 2012), I suggest, is precisely because actors see these models as providing access to some fundamental truth, even if (as will be discussed below) the concrete modelling practices themselves may be regarded as a ‘trick’.

Empirical ontology thus offers a useful way of viewing the politics of modelling that combines elements from both the modular and the practice-based perspective. On the one hand, modelling ontologies are political because specific enactments of them constitute objects in specific ways and not others. On the other hand, by focusing on diverging enactments of the same object, empirical ontology offers a model for action-based politics, drawing attention to the competing programs that may come with specific ways of enacting an object.

### The two modelling ontologies

In the case study of insurance, it is possible to distinguish between two modelling ontologies: an ontology of risk-neutral and of real-world probabilities. To see how these differ, it is useful to consider what financial models do. Financial models (and indeed the valuation models in insurance) seek to ascribe economic value to a series of future cash promises that are conditional on future events. To estimate the value of an instrument, the modeller typically seeks to ‘discount’ the value of future cash flows to compensate for ‘opportunity costs’ (or the benefits that could have been obtained if the money were invested in an alternative investment) and the uncertainty surrounding whether future cash flows will actually materialize (Deringer, 2017). Determining an appropriate discount rate is rather arbitrary, for it depends on judgments about how significant opportunity costs and uncertainty are. It requires forecasting, and especially in the context of economics the future is ‘fundamentally uncertain’ (e.g. Beckert, 1996).

The unknowability of the future is particularly problematic for derivative instruments like ‘financial options’, which provide their owners with the right, but not the obligation to buy (in the case of a call option) or sell (in the case of a put option) an underlying asset at a predetermined price. The value of these options depends entirely on the likelihood that the price of the underlying asset will exceed or be below the predetermined price agreed in the option contract. To value an option, in other words, practitioners may form a view on what the likelihood of all future possible outcomes will be, a view that may be informed by what Collier (2008) refers to as ‘archival-statistical knowledge’, but that will remain somewhat arbitrary nonetheless.

The peculiarity of no-arbitrage modelling is that it intends to provide a ‘synchronist’ alternative to archival-statistical financial modelling – an alternative that ‘promises to make all predictions redundant’ (Langenohl, 2018: 37). At the core of the most prevalent interpretation of no-arbitrage models is the concept that two different assets (or portfolios of assets) generating equivalent cash flows should be valued the same. If not, opportunities for ‘arbitrage’ would occur, allowing investors to make ‘riskless’ profits by simultaneously buying the cheaper asset (or portfolio of assets) and selling the dearer one. Due to the increased (decreased) demand for the cheaper (dearer) asset, prices would converge, making the arbitrage opportunity disappear. Hence, as modern finance theory posits, in an efficient market, opportunities for arbitrage should not exist, for they would immediately be arbitraged away by keen investors.

The Black-Scholes-Merton model for options pricing, which has served as an ‘exemplar’ from which other, more general models have been derived (MacKenzie, 2003: 855, 2006: 139), draws on the logic of no-arbitrage to establish a relation between an option contract – which gives its owner the right but not the obligation to buy (or sell) an underlying asset at a predetermined price – and its underlying asset. It is possible, the model suggests, to construct a portfolio comprised of the underlying asset and a ‘risk-free’ asset, which, if adjusted dynamically, could together mimic, or replicate, the pay-off structure of the option. In the context of well-functioning financial markets, the market value of such a replicating portfolio should be equal to the value of the option; if not, an arbitrage opportunity would arise, which would then be exploited by investors buying the option and selling the replicating portfolio as a ‘hedge’, as a consequence of which the price discrepancy would disappear.

Striking about the Black-Scholes-Merton model is that due to its widespread use actual market prices converged with those predicted by the model – at least momentarily so (MacKenzie, 2006, 2007; MacKenzie and Millo, 2003). The model has enjoyed, in other words, a great degree of ‘performative power’ (Svetlova, 2012), which may be attributed to its ‘high academic standing’, its practical utility, and its public availability (MacKenzie, 2007: 71). For many, these virtues derive from the fact that the ‘world in the model’ possesses specific ontological qualities. LiPuma, for instance, argues that the specificity of the Black-Scholes-Merton model derives from the fact that it ‘posits the existence of a specific socially imagined totality, *the market*, that renders the valuation problem mathematically tractable’ (LiPuma, 2017: 2, emphasis in original). Chiapello and Walter (2016: 164) suggest that no-arbitrage models imagine ‘a new world, the risk-neutral world’.

Thus, what sets no-arbitrage models apart from the traditional ‘real-world’ valuation models in finance (and, indeed, insurance) is that they are rooted in a specific set of assumptions about ‘what “the economic world” is made of’ – a distinct ‘ontology of probabilities’ (MacKenzie and Spears, 2014: 395, 398). No-arbitrage models rely on a calculation convention known as ‘risk-neutral pricing’, which, for those who are not a member of the no-arbitrage modelling culture tends to be rather difficult to grasp (MacKenzie and Spears, 2014). In a no-arbitrage model, the modeller constructs a world of expectations ‘in which all invested assets are assumed to provide the same expected rate of return, namely the risk-free rate, regardless of the risk of each specific asset’ (Chiapello and Walter, 2016: 164). The modeller achieves this by adjusting the probability measure that describes the likelihood of the spectrum of possible future price movements, so that the expected return of all assets equals that of the ‘risk free’ asset. The probabilities in the risk-neutral world are thus different from the probabilities assumed in a ‘real-world ontology’, which has some distinct advantages. Within the risk-free world, all assets can be valued by simply discounting the excepted future cash flows at the risk-free rate of interest.

In contrast to real-world probabilities, risk-neutral probabilities do not purport to represent the ‘true’ probabilities of particular events; nonetheless, they provide access to a reality to which the real-world probabilities cannot refer. The probabilities in the risk-neutral ontology are thus ‘simultaneously less real and more real than actual probabilities’ (MacKenzie and Spears, 2014: 400).

Conveniently, the risk-neutral ontology of no-arbitrage models allows for the ‘backing out’ of an ‘implied volatility’ when a market price of both the derivative and its underlying asset are known (Millo and MacKenzie, 2009). This enables a modeller who wants to price a newly traded derivative contract to derive a ‘market implied volatility from the prices on the underlying asset and a derivative that has the same underlying asset, but with a different strike price or maturity. In such cases, the modelling is (in ideal cases) non-arbitrary, as its value depends entirely on information derived from ‘market observables’, not on the ‘real world’ economic expectations of the modeller. This is why no-arbitrage models are often considered ‘objective’ and ‘scientific’: Two different no-arbitrage modellers should in theory arrive at the same price for an asset, regardless of the individual modellers’ expectations of what the future holds and so economists like Leon Walras perceived no-arbitrage type models as the only way to approach economics ‘scientifically’ (Langenohl, 2018). This, I suggest, is crucial for understanding the subterranean politics of no-arbitrage modelling.

## Modelling insurance

Modelling is a core activity in the everyday practice of life insurance. Life insurance contracts often extend into the distant future, yet insurers need to make decisions in the present regarding that future. To have a sense of how much profit will have been generated from a contract in the future, modellers may, for instance, want to predict the likelihood that a customer will survive beyond a given age, how high future administrative expenses will be, the likelihood that customers ‘lapse’, and the rate of interest that the company is likely to earn on the premiums that it invests. The products of such modelling exercises may inform an insurer’s decisions, such as about the pricing of its products, its bonus policy (how much of a company’s surplus it will distribute across policyholders in the case of with-profits insurance), and its ‘reserving’ practices (the amount of assets it sets aside to back up future claims). Similarly, insurers’ valuations are ‘obligatory passage points’ (Callon, 1986) for ‘solvency’ assessments (estimations of whether a firm will have sufficient assets to pay future policyholder claims).

Throughout much of the twentieth century, the modelling of life insurance fell squarely within the professional jurisdiction of the actuarial profession. Actuaries applied their archival-statistical knowledge of mortality to make projections of future profitability. The actuarial modelling of insurance, however, is considered to involve not only statistical knowledge, but also ‘expert judgment’ (Alborn, 1994; Porter, 1995). This is because the various forms of uncertainty involved. National mortality statistics, for instance, may not be representative of a company’s more specific population of policyholders, which causes uncertainty surrounding ‘true mortality’.^1^ Thus, while much of the uncertainty surrounding insurance on individual lives may be diversified away by spreading ‘risk’ across a larger population, several forms of uncertainty remain. In applying their ‘expert judgment’, the traditional actuarial approach requires the modeller to estimate the parameters of the model *prudently*. By deliberately over- or underestimating model parameters, the modeller seeks to ensure that the representation of a firm’s financial position remains on the safe side of things.

The authority of traditional actuarial modelling thus relied on the legitimacy of actuarial judgment. In the late 1990s and early 2000s, however, the status of the actuarial profession and its approach to modelling started to decline, while, at the same time, modern finance theory had become increasingly authoritative. In 1997, for instance, two of the three authors of the Black-Scholes-Merton model received the Nobel Prize in economics (Fischer Black died in 1995 and was therefore not awarded the prize). The decisive factor in fostering the appropriation of no-arbitrage models in the context of insurance, however, was the fact that the UK’s Financial Services Authority required insurers to use ‘the same techniques as are used by banks and other participants in the capital markets to quantify [their liabilities]’ for solvency calculation purposes (Davies, 2002). While the responsibility to regulate insurers’ behaviour historically had been delegated to the actuarial profession, the declining status of the profession contributed to the displacement of this responsibility to the increasingly authoritative market-consistent models.

## The four stages of migration: From actuarial modelling to market-consistent modelling

I identify four stages in the shifting distribution of authority. In the first stage, actuaries considered financial economics as a potential resource for taking into account the financial risk embedded in insurance contracts, but rejected the usefulness of no-arbitrage models for their limited practical utility. In the 1970s, ‘financial risk’ became an increasingly central epistemic problem within the actuarial profession, partially due to increased financial market volatility and partially due to the types of products that insurers were selling. By the late 1970s, not long after the publication of the Black-Scholes-Merton model, various actuaries had already noted the similarity between insurance guarantees and financial derivatives such as options (Collins, 1982; Corby, 1977; Ford et al., 1980). A maturity guarantee (promising the policyholder a guaranteed benefit at the maturity of the contract), for instance, could be construed as being similar to a stock-index option. It was thinkable, these actuaries noted, to decompose an insurance contract into its constitutive elements and to value these elements independently using no-arbitrage modelling. Some of these early investigations, for instance, concluded that the ‘theoretical approach’ of no-arbitrage modelling ‘does seem to have serous practical disadvantages because it depends upon several underlying assumptions’ (Ford et al., 1980: 112).

In the second stage, the potential role of financial economics in insurance became more controversial. A new generation of actuaries emerged whose members became avid proponents of the financial economic approach to valuation, some of whom started using techniques from modern financial economics to evaluate the economic value of insurance undertakings. As one of its members writes, these were actuaries ‘who had independently developed expertise in financial economics and who worried that the British actuarial profession was dangerously behind other financial professionals’ (Turnbull, 2017: 216). The emergence of the financial-economic minded actuaries gave rise to significant friction within the profession, which manifested itself in various ways. When proponents of no-arbitrage modelling presented their ideas at the profession’s sessional meetings, for instance, the discussion tended to get heated. As one actuary recalls: ‘There was a real backlash of “you can’t say that, because if you’re right everything that we’re doing is wrong”’ (interview CK).

Frictions also surfaced in debates surrounding the actuarial examination system. Some of the early proponents of financial economics had difficulties passing their professional exams, because, as interviewee CK remembers, ‘the examinations at the time were teaching things which from a pure finance perspective didn’t make sense’. Most opted to answer the questions as they thought they were expected to answer them, just to obtain their actuarial qualification. One actuary, however, refused to do so. In an article published in the profession’s magazine *The Actuary*, Andrew Smith explained that he preferred to wait for the exam system to be reorganized. ‘Why lose sleep now for the sake of an exam system that is obviously in chaos?’, he asked (Smith, 2005). In this second stage, the debate on valuation had opened up, but proposals to adopt a finance perspective on insurance met with strong resistance.

The third stage occurred in the early 2000s, when the actuarial profession became embroiled in a crisis of legitimacy. Towards the late 1990s (and continuing in the early 2000s), many UK life insurers were in financial trouble. The problems arose as a consequence of persistently declining interest rates and sustained improvements of life expectancy above actuarial expectations. Companies that had sold large amounts of with-profits endowment policies containing ‘guaranteed annuity options’ (GAOs) were particularly affected. These options gave policyholders the right, but not the obligation, to convert lump sum pension benefits into an annuity at a predetermined rate, providing a regular stream of income until maturity or death, whichever would come earlier. When the GAO policies were sold (mostly in the 1970s and 1980s), the rates guaranteed were much lower than market rates. Companies would sell, for instance, GAO policies with an option to convert a lump sum endowment into an annuity that would provide annual payments of £10 per £100 cash from age 70 onwards, while market rates were (as in the 1980s) typically well above £15 per £100 cash. Because of this, insurance companies undertook little effort to maintain explicit reserves in case the guarantees would ‘bite’ (O’Brien, 2006).

By the late 1990s, however, it became increasingly clear that interest rates had declined (and hence market annuity rates had dropped) to such an extent that many of the GAO policies started to bite. Declining stock markets in the early 2000s put firms under even more pressure. Particularly affected was the Equitable Life Assurance Society, the UK’s first life insurer to use actuarial modelling in its pricing. The company had competed aggressively to increase its market share in the pension annuities market, but it had done so at the expense of offering what would turn out to be very costly guarantees.^2^ In the early 2000s, after a court case, the guarantees proved to be too costly, and facing substantial shortfalls the company shut down for business (O’Brien, 2004, 2006). The affair turned into a major scandal, with estimated policyholder losses totalling £4.1bn, of which £1.5bn were covered by the government (HM Treasury, 2016).

The collapse of Equitable Life led to substantial critique of actuarial practice. A Treasury investigation led by Lord Penrose concluded that the ‘regulatory returns and measures of solvency applied by the regulators did not keep pace with developments in the industry … regulatory solvency became an increasingly irrelevant measure of the realistic financial position of the Society’ (Penrose, 2004: 727). The regulatory regime allowed insurance companies substantial freedom in deciding how to measure their liabilities, but in practice this implied the use of ‘traditional’ actuarial methods. These deterministic methods were considered useful for determining how to distribute bonuses, but less so to estimate the cost of guarantees and non-contractual promises (such as prospective terminal bonuses). While stochastic methods to estimate the cost of such guarantees were available since the 1980s, these were hardly used, mainly because actuaries argued that the discretionary bonus system provided them with sufficient leeway to maintain the fund’s solvency. A review of the profession, sparked by the Penrose inquiry, concluded that the profession’s ‘professional standards … have been weak, ambiguous or too limited in range, and [were] perceived as influenced by commercial interests’ and ‘that [it] has been too introspective, not forward-looking enough and slow to modernise’ (Morris, 2005: 15). The guaranteed annuity option crisis, in other words, contributed significantly to the weakening of the profession’s jurisdictional claims (Collins et al., 2009). Although it is unlikely that, without this crisis of legitimacy, the actuarial profession would not have drawn on financial economics at all, it nonetheless fostered the diffusion of no-arbitrage modelling in the world of insurance.

The fourth and final stage occurred from the mid-2000s onwards, when the Financial Services Authority started requiring insurers to quantify the value of their liabilities using market-consistent models. This shift should be understood within the international context. To foster the integration of European insurance markets, the European Commission had decided to harmonize European solvency regulation, and to bring it in line with solvency regulation for banks (Swain and Swallow, 2015). Similarly, international accounting standard setters were keen to develop a ‘fair value’ accounting regime, which would reflect the true economic value of insurers’ assets and liabilities. Part of the appeal of banks’ no-arbitrage models, according to Craig Turnbull, is their alleged objectivity:

I think one of the fundamental attractions to adopting these approaches … was this idea that you could get to an objective measure of these costs in a way that removed actuarial judgment … or strange actuarial assumptions that no one else understood. You know, you mark-to-market and there’s market prices. You use them. And that was your price, and it would be this objective single answer. (Turnbull interview)

Initially, the UK’s Financial Services Authority intended to wait with reforming the domestic systems for European regulations to come in place. However, when it became clear that such reforms would take a long time to crystallize (Solvency II, as the framework is called, was implemented only in 2016; a ‘fair value’ accounting standard for insurance liabilities was published only in 2017 and at the time of writing has yet to be implemented) the Financial Services Authority decided to ‘front run’ these developments by implementing what they called (admittedly somewhat confusingly) the ‘realistic balance sheet’ regime. Insurers were now supposed to use not the traditional ‘real-world’ actuarial models, but market-consistent ones.

The application of no-arbitrage models in the context of insurance was far from straightforward. Although the insurance guarantees were in many respects similar to financial options, there were also many obvious differences. As interviewee CK remembers, ‘a lot of the stuff that … comes out of finance and banking isn’t directly applicable, you know, because the products are different’. When the Financial Services Authority decided to move to a market-consistent valuation regime, it was aware of this. In an early report on the subject, for instance, it referred to ‘realistic reserving methods’ as a ‘developing art’ (Financial Services Authority, 2003: 23). The fourth ‘moment of translation’ (Callon, 1986), in other words, encompassed the construction of ‘market-consistent’ models that were based in the same ontology of banks’ no-arbitrage models, but were structurally and (in some specific implementations of it) conceptually distinct.

## The ‘rough edges’ of market-consistent modelling

The construction of insurers’ market-consistent models involved the analogical extension of existing no-arbitrage models and bricolage. Since the 1970s, actuarial valuation models were increasingly run on computers, either in Excel or specialist valuation software built by actuarial consultancies. In the case of a traditional actuarial valuation, the modellers would simply calculate the value of the liabilities within a single ‘prudent’ scenario (comprised of point-based estimates for the different risk parameters). In the case of market-consistent modelling, however, insurers typically run the valuation software for a range of different ‘risk-neutral’ scenarios.^3^ The value of a single liability is then obtained by averaging the value of the liabilities across the different scenarios. The scenarios are produced not within the valuation model, but with a different piece of software, a risk-neutral economic scenario generator, which projects a range of economic variables under the assumption that their expected rate of return is equal to the risk-free rate of interest. The outputs of the economic scenario generator were simply fed into the already existing valuation software.

The main innovation was thus to develop an economic scenario. This, according to my interviewees, happened rather haphazardly. A modeller who worked at one of the main model providers at the time explained: ‘What we were basically doing was picking up those models that were already used for valuation problems [in banking] and … putting them all together in one large model… almost sort of glue them together into one model’ (interview CF). Another modeller confirmed that the model providers, at least initially, ‘patched together off-the-shelf banking models that you could get from textbooks … [but] that aren’t really supposed to work together’ (interview CJ). While the no-arbitrage models served as an exemplar for insurance liability valuation, knowledge for how these models were to be applied in the context of insurance developed in parallel with the models themselves. Indeed, interviewee CK recalls that ‘everybody was learning at the same time’, including supervisors.

The implementation of market-consistent modelling was complicated by what Andrew Smith referred to as the ‘rough edges’ of market-consistent modelling. These rough edges emerged when differences between the idealized world posited by no-arbitrage modelling and the concrete markets to which the models refer became problematized. In the development of insurers’ market-consistent models, three main types of rough edges can be distinguished: (1) those due to differences between the objects to be modelled, namely insurance contracts and financial instruments; (2) calibration problems that emerged from the limited range of financial instruments that are actually traded in financial markets; and (3) the emergence of phenomena that seem to influence the value of an insurance liability, but that cannot be accounted for within the world of the model.

The first two of these epistemic challenges arose because insurance liability valuation was considered an ‘imperfect market problem’. In the idealized world of no-arbitrage models, markets are complete. In a complete market all instruments needed to ‘hedge’ the risks of a given asset are available so that a ‘replicating portfolio’ may be constructed. When valuing insurance liabilities, however, this may not always be the case. The actuary Angus Macdonald described the problem as follows at one of the profession’s sessional meetings:

We are not dealing with a market in which all the instruments are traded both ways. You cannot decide arbitrarily to buy or sell a life insurance policy. Also, the very notion of doing so only considers the pure investment part of the risk, and ignores all other aspects of a with-profits policy, such as the pooling across generations and the smoothing. (Macdonald in Hare et al., 2000: 208)

The risk-neutral world of no-arbitrage models can only take account of the financial dimensions of an insurance liability. The relation between non-financial risks and the value of the instrument, however, cannot be described in terms of arbitrageable relations to other financial market assets. Instead, market-consistent modellers are expected to make an estimate of those risks. The estimation of insurance risk parameters like mortality, longevity or lapse risk, however, brings the modeller back into the world of ‘real-world’ expectations.^4^ This has important consequences for the perceived objectivity of the model. As Andrew Smith put it, the need to come up with a point-based estimate of insurance-related risks means that ‘you could, potentially, have two models both claiming to be market consistent and giving different answers’ (Smith in the discussion of Sheldon and Smith, 2004: 624).

A second epistemic problem was due to the limited range of financial instruments that are actually traded in financial markets. The length of life insurance contracts, for instance, often exceeds those of the derivatives contracts that investment banks are willing to sell. To calibrate a market-consistent model, however, modellers need information about the implied volatility, for instance, of a stock-index put option maturing in 20 years’ time.^5^ Modellers may obtain such information by asking banks for price quotes, even if the latter are not willing to trade at those prices. This is how a modeller explained his firm resolved this problem:

[T]he shorter term options have more weight in the calibration, but when we look at the longer-term options, we’re essentially extrapolating towards something which is informed by our real-world views. Because we don’t have … market information … you’re sort of constructing a pseudo option-implied vol [volatility] in the long term … using real-world assumptions to inform what an implied volatility might look like. (Interview CF)

The modelling of long-term liabilities, in other words, involves expectations about the hypothetical price at which investment banks would be willing to trade long-dated options, a hypothetical price that is derived from extrapolating the implied volatility of shorter dated options and from economic expectations about what the long-dated implied volatility might look like.

While the above two epistemic problems were considered practical in nature, a third category of epistemic problems was considered more fundamental. These problems concern phenomena that seem to influence the value of insurance liabilities, but that cannot be accounted for in what one of my interviewees described as the ‘theoretically pure’ world of no-arbitrage modelling. This is because practical implementations of the ontology of no-arbitrage require idealizations that make the models mathematically, or, in most cases, computationally tractable (see for a list of idealizations identified by actuaries: Sheldon and Smith, 2004: 590). No-arbitrage models, for instance, ignore the effect of tax regimes on the valuation of insurance liabilities. Similarly, no-arbitrage modellers assume that instruments have a single market price, while in reality instruments have both a ‘bid’ and an ‘ask’ price (which is indeed an important source of profit for the banks buying and selling derivatives from and to investors like insurance companies). While actors may not necessarily consider such idealizations problematic, the important point is that they may be *problematized* at any point in time, thus providing interested actors with a resource to challenge the veracity of the model.

## Seeing the world in market-consistent terms

So far, I have described the conditions that paved the way for no-arbitrage models to migrate, and the construction of market-consistent models. In this section, I focus on the ontological politics of insurance modelling by looking at how market-consistent models affected the reality of insurance.

The most immediate consequence was that the modelling of insurance contracts as bundles of financial instruments contributed to insurers’ changing investment behaviour. Because insurers started *enacting* their insurance policies differently (namely, financially), this also had consequences for how insurers *acted upon* them. In a market-consistent model, the question is not: What will the future look like? The question is rather: What is the cost of investing your assets in such a way that uncertainty about your ability to meet your liabilities is reduced to a minimum. In the late 1990s, insurers rarely used derivatives to manage their investment-related risks, simply because, as one actuary suggested, ‘it wouldn’t have occurred to you to go and get an interest swap or an interest rate swaption because you wouldn’t have thought of it like that’ (interview EA). Indeed, in 1996, the total notional amount of derivatives outstanding amounted to a meagre £800 million (Hardwick and Adams, 1999).^6^ Since the early 2000s, however, various investment banks have increasingly started hiring actuaries to help sell the derivatives. In 2010, Standard Life alone reported having more than £57 billion in notional value of derivatives outstanding, with the figure increasing to £120 billion in 2016. As insurers started to enact insurance contracts ‘financially’, their management through derivatives strategies became a ‘thinkable’ one.

The financial enactment of insurance contracts thus had an important influence on how practitioners perceived and understood insurance. The specific way in which the risk-neutral ontology was operationalized also mattered. As various modellers agreed, initially there was some confusion around the meaning of risk-neutral scenarios. The assumption that all assets generated a risk-free rate of return, which the market-consistent modellers perceived simply as a ‘neat trick’, was difficult to accept for those less versed in no-arbitrage pricing. For strategic purposes, Andrew Smith at Deloitte therefore developed a model that was mathematically consistent but conceptually different from the risk-neutral models developed by its competitors. Risk-neutral pricing works because the effects of assuming that all assets earned a risk-free rate of interest is proportionally offset by the assumption that all future cash flows should be discounted at the risk-free rate. This, however, was not a mathematical necessity. In effect, a no-arbitrage modeller may pick any set of probabilities (thus affecting the expected rate of return on an asset) as long as the discount factor is adjusted appropriately. A modeller can start from a real-world probability measure (rather than a risk-neutral one) and should, in theory, arrive at the same answer as a risk-neutral model when s/he uses a ‘stochastic discount factor’ – consisting of a risk-free component and a ‘deflator’ component – to discount the value of future cash flows. This is what Smith sought to do. Although mathematically more challenging to calculate, Smith’s deflator model would be easier to interpret. As he put it, ‘the deflator was a way of explaining, well, this is how you get from a realistic-looking model to something that replicates market prices’ (Smith interview).

Initially, the Smith Model was intended as ‘a model that could do both real-world and risk-neutral within the same set of scenarios’ (Smith interview). While companies would use the risk-neutral scenarios to value their liabilities, the real-world scenarios were considered more appropriate for risk management purposes. The Smith Model would use the ‘deflator’ function as a ‘change of measure’ to switch between the two worlds. While switching between worlds worked in theory, in practice ‘there’s some subtlety to this’ (Smith interview). The theoretical possibility of switching between model worlds depends on the assumption that the real-world and risk-neutral models are in all other respects the same. In practice, however, this assumption can be challenged. It is well-known, for instance, that ‘historically, option implied volatility has been higher than the corresponding real-world volatility’ (interview CF). This means, as Smith put it, that ‘a model which would replicate market prices was not, in mathematical terms, absolutely continuous with respect to any credible model of real-worlds’ (Smith interview).

The discontinuity between a market-consistent model and a ‘credible’ real-world model, as interviewee CF explained, has practical implications:

If you use the option-implied volatility, you’d probably be overstating … the real-world volatility. If you use the real-world volatility, you would underprice options. So if you want to use the same model for two different things, constraining yourself by the theoretical straitjacket means you’re actually doing neither of those things particularly well. (Interview CF)

One area where the calibration of the model ‘really mattered’, according to Smith, is deciding when to hedge the risks of a specific book of liabilities. As Smith explained:

if you are an insurer and you’ve written some liabilities that had guarantees and you were considering whether to hedge or not, if you use … [a] model calibrated to market prices of options, then hedging always looked like a fantastic idea, because you got rid of all these risks of things going wrong, and you would just pay a fair price for the option, whereas if you used the historic calibration it’s much more a trade-off, because you think the volatility is going to be 15%, the market is charging 20%, but … when you’ve paid that 20, you’ve got some peace of mind… [Y]ou’re no longer exposed to your model being wrong. (Smith interview)

Even though the two approaches to market-consistent modelling were mathematically continuous in theory, they were not in practice. The choice for either approach was not only ‘cosmetic’, but also might have important implications for decision-making. The point here is that what seems to be exclusively a technical issue may influence how a problem is construed (and thus how it is addressed).

Market-consistent modelling did not only influence insurers’ *internal* decision-making. It also affected how the company was perceived by *external* actors such as investors. As one of my interviewees explained:

In the old days … [investors in life insurance] didn’t really understand anything about what went on underneath the bonnet of the car. They just knew that the car drove off at a fairly steady pace in the right direction …. [C]ompanies like Prudential were invested in because they threw off this steady dividend string. Nowadays that isn’t the way it works. With-profits is a much smaller part; it doesn’t produce a consistent profit stream, because of the lower interest rate environment, and so forth. And they’re now simply looking at reporting profits on the short-term. And so the valuation methods take an excessive priority, because they drive investor sentiment … the world is being driven by the valuation practice. (Chamberlain interview)

In what Chamberlain describes as the ‘old days’, life insurance was a ‘black box’ for investors; companies’ performance could only be measured by investors through the dividends that they generated. Whether the ‘engine’ underneath the bonnet of the car was sound and whether a company would be able to sustain such dividend streams was up to the actuary. In contemporary insurance, however, insurers periodically produce representations of the processes that take place underneath the bonnet of the car through market-consistent valuations (and risk capital calculations) and leave investors to decide whether the profit streams promised by the insurer are ‘credible’.

## Problematizing and politicking

I have shown how market-consistent models may be considered political in terms of their consequences. When models become an ‘obligatory passage point’ for political decision-making, however, they may also become the object of ‘politics as activity’ (Brown, 2015; Sundberg, 2007). Precisely because models are idealized representations of reality (and thereby constitute a specific enactment of reality), they may be influenced by political forces. In political controversies around climate change, for instance, actors may deploy strategies to delay scientific closure by questioning the idealizations that underpin the modelling work (Edwards, 2010). Pointing at the idealized and constructed nature of model output may sow doubt about the model’s overall veracity and authority. When becoming obligatory passage points for policymaking and regulation, models may quickly become the object of politicking.

The problematization of insurers’ market-consistent models especially revolved around the discounting rules, which prescribe how insurers must translate future cash flows into a ‘present value’. Traditional actuarial valuations discounted liabilities at interest rates that reflected expectations about a company’s future investment income on ‘risky’ assets (Patel and Daykin, 2010). What interviewees referred to as a ‘theoretically pure’ market-consistent regime, however, requires ‘risk-free’ discounting.

One of the rough edges of risk-free discounting is constituted by the fact that ‘there is no such thing as a risk-free rate’, as one of my interviewees put it. ‘It is a theoretical construct. It’s not something that exists in reality’ (interview BC). Although interest rates on government bonds are considered good proxies for the ‘risk-free’ yield curve, ‘even sovereigns have risk’ (interview BC). Interest rate swaps are typically considered riskier than government bonds, which is reflected by the fact that their implied interest rates are higher. For some, this meant that government bonds were the better reference asset for the risk-free curve (see Sheldon and Smith, 2004; Dullaway and Needleman, 2004). In the context of European capital regulation (Solvency II), however, the use of government bonds was considered problematic. Interest rates on government bonds tend to diverge across member states of the Eurozone, which would imply that the same euro-denominated insurance liability would be valued differently across different jurisdictions – an outcome that would be politically unacceptable to countries whose government bonds have relatively low interest rates. This political consideration proved decisive; the risk-free curve for euro-denominated liabilities is based primarily on interest rate swaps, not on government bonds.

Another related issue – already referred to above – is that the maturity of insurers’ liabilities may exceed those of the interest-rate swaps from which the risk-free discount curve is derived. This issue came up again the European context, where different member states were to agree on a common approach. As interviewee CA noted, some European insurers ‘sell pension policies to 30-year olds’ with guarantees that may well extend 60–70 years into the future – but the longest maturity of German government bonds is 30 years. Thus the time horizon of insurers’ liabilities may well exceed that of financial markets. For insurers to discount their long-term liabilities at a market-based estimate of the risk-free rate thus requires the extrapolation of the discount curve into the future, for which there is no uniquely accepted method.

One approach would have been simply to use as much market information as possible and simply to extrapolate market rates into the future at a constant level. In the present era of low interest rates, however, this would have meant that insurers’ long-term liabilities would be discounted at rather low interest rates that, as Hare put it, ‘could just create solvency issues for a number of foreign insurance companies, and potentially serious issues for some’. The construction of the risk-free curve thus became a ‘big political negotiating point’, which some argued played a big part in delaying the implementation of the European framework (interview CD).

**Figure 1.**
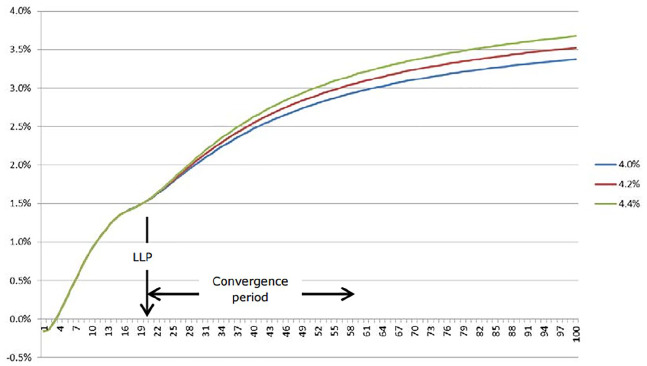
A graphical representation of the risk-free curve that insurers are required to use for European insurance capital regulation (Solvency II). The figure shows what the curve would look like for three different values of the ultimate forward rate. The size of the ultimate forward rate is respectively 4.4% (green), 4.2% (red), and 4.0% (blue). Source: EIOPA (2016).

Instead of simple extrapolation, the European Insurance and Occupational Pensions Authority (EIOPA) eventually decided on a different approach (see Figure 1), which divides the risk-free curve in two parts. One part is directly derived from market interest rates on interest rate swaps and government bonds; it covers the relatively short-term maturities up to the last liquid point (LLP), which is the point at which EIOPA treats market information as relevant. The second part covers the curve from the last liquid point to a maturity of a 100 years by extrapolation of the curve towards an ‘ultimate forward rate’ – the rate to which the curve is assumed to converge at infinite maturity. EIOPA claims that the ultimate forward rate is derived from archival statistical knowledge of past interest rates. At the time this research was conducted, however, many perceived the ultimate forward rate as artificially high and the last liquid point (typically set at 20 years as indicated in Figure 1) as unnecessarily short-term.^7^

The result is a curve that is visibly higher (implying a lower value for the discounted liabilities) than would have resulted from a relatively simple extrapolation of market rates. Some of my interviewees argued that the choice of methodology was ‘overtly political’ to accommodate for some large continental European insurers (Smith interview). The choice of methodology, however, also had important implications for the valuation of domestic insurers. Take for instance the last liquid point, which limits the amount of market information that is used. According to Smith,

A theoretically pure perspective will be to say: We’ll just use the market curve. Why are we kidding ourselves that the market prices beyond 20 years are perfectly relevant for valuing assets [because assets are simply accounted for at ‘market value’], but not for valuing liabilities [because liabilities are discounted at an extrapolated rate of interest that exceeds market interest rates]?

This discrepancy also raised questions about insurers’ ‘matching’ strategies, which allows them to ‘buy-and-hold’ assets without having to sell them for liquidity needs: ‘How do I do my asset and liability matching when I’m pretending that I’m getting yields which I’m not?’ (Hare interview). Thus, while the notion of market-consistency requires insurers to use as much ‘market information’ as possible, many insurers face incentives to engage in politicking to reduce the amount of market information that is actually used.

What this example ultimately shows, is that the theoretical ‘rough edges’ of models provide space for politicking. Interested actors may strategically seek to make use of a model’s rough edges to influence its design in specific ways. They must do so, however, in ways that are conceptually defensible even if methodologically rather arbitrary and, according to some, problematic.

## Conclusion

The purpose of this paper was to consider the problem of model migration from the perspective of empirical ontology. I argued that in the case of life insurance valuation, and indeed in finance more generally, two modelling ontologies can be distinguished: a ‘real world’ modelling ontology, which assumes that the value of financial instruments is determined by real probabilities may be approximated through historical analysis and expert judgment; and a ‘risk neutral’ ontology, which assumes that the value of financial instruments is determined through arbitrage relations, and may be approximated through risk-neutral pricing. Even if risk neutral probabilities themselves are merely regarded as a trick, they are nonetheless understood to provide access to the deeper truth that the price of an asset as determined by the logic of no-arbitrage. The shift in the modelling of insurance from one ontology to another, which was made possible by the declining legitimacy of traditional actuarial valuation methods, had some important implications. By enacting insurance contracts financially, they were also increasingly treated as financial instruments: as bundles of risk that can be bought and sold in liquid capital markets.

Distinguishing between the two ontologies allows me to make two main points concerning the politics of modelling that have relevance beyond the specific case of insurance valuation. First, the different modelling ontologies are entangled with the distribution of authority. When the legitimacy of actuarial expertise was compromised, so were the models that explicitly required expert judgment. The risk-neutral ontology of modern finance’s no-arbitrage models provided an alternative: it imagines financial markets as an idealized calculative space that promises to do away with prediction and instead analyses financial value as a synchronous problem, thus promising to reduce the discretionary space afforded to the actuary. To understand why some models are so salient, it is necessary to take into account how a model articulates not just with concrete organizational goals (e.g. Millo and MacKenzie, 2009), but also with the broader social, political and technological ecology (such as the distribution of authority) in which it is deployed.

Second, models have ‘rough edges’, which means their concrete specification may potentially become the object of political negotiation at all times. These rough edges are constituted by a ‘gap’ between the rules or idealizations that come with a modelling ontology and its application to concrete cases (cf. Barnes, 1982; Bloor, 1997). Thus, even if the risk neutral ontology promised to do away with judgment, it could not do so entirely due to the rough edges of no-arbitrage modelling. The idealizations that help make the valuation problem mathematically or computationally tractable left the models vulnerable to the critique that they were not ‘realistic’. When the idealizations were considered to have problematic consequences, as in the case of the discounting curve, modelling overtly became the object of political negotiation. The valuation models that insurers use today, in other words, ‘have politics’ in the sense that they are shaped by past political compromises.

## Notes

1. There is also uncertainty about whether past mortality statistics provide a relevant indication of future mortality experience. This epistemic problem, however, only became prominent at the start of the twenty-first century.

2. Equitable initially was an important supplier of such pensions policies to the Federated Superannuation Scheme for Universities, but in response to losing this business in 1970, started to compete aggressively ‘in the endowments and pensions field’ (Corley 2001: 3, Appendix 3).

3. This is a different strategy then the one originally envisioned: Instead of decomposing the insurance contract into its constitutive elements and valuing each of them separately, an economic scenario generator produces a set of ‘risk-neutral scenarios’ that are then fed into a valuation model to value a policy in its entirety. The decompositional strategy was used, nonetheless, for a different type of market-consistent model: the replicating portfolio model. This model was understood as technically inferior to the economic scenario generator approach, but, because it was much quicker, some considered it to be more useful for management purposes.

4. Similarly, the guarantees written by insurers are constructed as options on an underlying investment fund. This investment fund may contain different assets, the ‘dependency’ between which matters for the volatility of the fund as a whole, and thus the price of the guarantee. The dependency between assets (or other sources of risk) thus brings the modeller into the realm of expectations too.

5. In ‘frictionless’ markets, according to modern finance theory, this would not be necessary. Within such a world, the implied volatility on options of different maturities should be the same. In the real world, however, this assumption does not hold. The implied volatility of longer-dated options, for instance, tends to be higher than those of shorter-dated options. As a consequence, ‘implied volatility’ is commonly used in financial markets to express and compare the relative price of options (see MacKenzie, 2006).

6. The notional amount of derivatives outstanding is a measure that tends to overstate the significance of derivatives, for it measures the value of the assets on which the derivatives are based rather than the value of the derivatives themselves.

7. EIOPA (2016) has since then proposed to derive the ultimate forward rate on an ongoing basis. As before, the derivation was based on central banks’ inflation target (different for different currencies) and a historical average of real rates. For euro-denominated liabilities, the new ultimate forward rate was calculated at 3.65%, significantly lower than the previous ultimate forward rate. To limit the impact on the balance sheets of companies with long-term guarantees on their books, the rate of change was capped at 15 basis points annually (which meant, e.g. that in 2018 – the first year in which the new methodology was applied – the ultimate forward rate was 4.05%).
